# Harzianic Acid Activity against *Staphylococcus aureus* and Its Role in Calcium Regulation

**DOI:** 10.3390/toxins15040237

**Published:** 2023-03-24

**Authors:** Alessia Staropoli, Paola Cuomo, Maria Michela Salvatore, Gaetano De Tommaso, Mauro Iuliano, Anna Andolfi, Gian Carlo Tenore, Rosanna Capparelli, Francesco Vinale

**Affiliations:** 1 Institute for Sustainable Plant Protection, National Research Council, 80055 Portici, Italy; mariamichela.salvatore@unina.it; 2 Department of Agricultural Sciences, University of Naples Federico II, 80055 Portici, Italy; rosanna.capparelli@unina.it; 3 Department of Chemical Sciences, University of Naples Federico II, 80126 Naples, Italy; gaetano.detommaso@unina.it (G.D.T.); mauro.iuliano@unina.it (M.I.); andolfi@unina.it (A.A.); 4 BAT Center-Interuniversity Center for Studies on Bioinspired Agro-Environmental Technology, University of Naples Federico II, 80055 Portici, Italy; frvinale@unina.it; 5 Department of Pharmacy, University of Naples Federico II, 80131 Naples, Italy; giancarlo.tenore@unina.it; 6 Department of Veterinary Medicine and Animal Productions, University of Naples Federico II, 80137 Naples, Italy

**Keywords:** calcium, chelation, fungi, secondary metabolites, *Staphylococcus aureus*, *Trichoderma*

## Abstract

*Staphylococcus aureus* is a Gram-positive bacterium, which can be found, as a commensal microorganism, on the skin surface or in the nasal mucosa of the human population. However, *S. aureus* may become pathogenic and cause severe infections, especially in hospitalized patients. As an opportunistic pathogen, in fact, *S. aureus* interferes with the host Ca^2+^ signaling, favoring the spread of the infection and tissue destruction. The identification of novel strategies to restore calcium homeostasis and prevent the associated clinical outcomes is an emerging challenge. Here, we investigate whether harzianic acid, a bioactive metabolite derived from fungi of the genus *Trichoderma*, could control *S. aureus*-induced Ca^2+^ movements. First, we show the capability of harzianic acid to complex calcium divalent cations, using mass spectrometric, potentiometric, spectrophotometric, and nuclear magnetic resonance techniques. Then, we demonstrate that harzianic acid significantly modulates Ca^2+^ increase in HaCaT (human keratinocytes) cells incubated with *S. aureus*. In conclusion, this study suggests harzianic acid as a promising therapeutical alternative against diseases associated with Ca^2+^ homeostasis alteration.

## 1. Introduction

Calcium is a universal intracellular messenger that regulates different cellular activities. It is responsible for controlling cell development and proliferation, as well as fundamental processes such as learning, memories, and muscle contraction [1]. Ca^2+^ signaling and regulation has been widely described in eukaryotic cells due to its involvement in above-mentioned cellular processes [2,3]. Given this scenario, Ca^2+^ signaling is highly regulated and its concentration tightly controlled in various cell compartments [4,5]. For this reason, if slightly exceeding, Ca^2+^ ions may become toxic and stimulate cell death, thus increasing the risk to develop severe pathologies [6]. External stimuli may induce significant changes in cell calcium concentration. It has been reported that bacteria are one of the biotic factors contributing to Ca^2+^ homeostasis alteration, for example, through the release of pore-forming toxins (PFTs) [7].

*Staphylococcus aureus* is a Gram-positive bacterium that, although commonly considered a commensal, is a major cause of several human infections (e.g., pneumonia, endocarditis, and medical-device-related), including skin and soft tissue infections (SSTIs) [8]. *S*t*aphylococcus aureus* is able to colonize skin and nasal mucosa without causing any symptoms. However, when colonizing immunocompromised individuals, *S. aureus* may build an infection [9,10]. In order to colonize and infect the host cell, *S*. *aureus* expresses virulence factors such as hemolysin A, a PFT that can form pores and insert into host cell membranes, resulting in a perturbation of calcium levels [7,11]. Alterations of Ca^2+^ concentration, amongst other pathogenesis mechanisms, could mediate bacterial adherence, following its incorporation into the host cells [7,12,13].

Starting from the discovery of penicillin, the interest in natural products has increased, since microbial strains play a key role as major sources of secondary metabolites for drug discovery and application in both medical and agricultural fields [14,15,16]. These secondary metabolites mediate interactions with plants, microbes, cells, and tissues and are involved in the effects on plants or other organisms [17,18]

Among fungal microbes, *Trichoderma* is the genus recognized as a model to study plant–microbe interactions [19,20]. Therefore, selected strains of *Trichoderma* are broadly commercialized for crop protection and production. [21]. Several strains of *Trichoderma* are also exceptional producers of secondary metabolites with a wide range of biological activities (e.g., antibiosis, plant growth promotion, induction of systemic resistance, transport of metal cations, etc.) [22,23].

Among these bioactive metabolites, there is harzianic acid (HA), a tetramic acid derivative that has demonstrated remarkable biological activities, including antimicrobial activity (e.g., *S. pseudintermedius*, *Rhizoctonia solani*), plant growth promotion (i.e., tomato, olive drupes), and affinity to ferric and other divalent metal ions [24,25,26,27,28,29,30,31].

In this context, harzianic acid may represent a valid therapeutical strategy to prevent detrimental diseases, having shown chelating properties towards bivalent cations [30,31]. The aim of the present study is to demonstrate the affinity of HA for Ca^2+^ ions and its ability to affect calcium mobilization in HaCaT cells upon *S. aureus* infection.

## 2. Results and Discussion

### 2.1. Harzianic Acid Inhibits Staphylococcus aureus Growth

**Figure 1:**
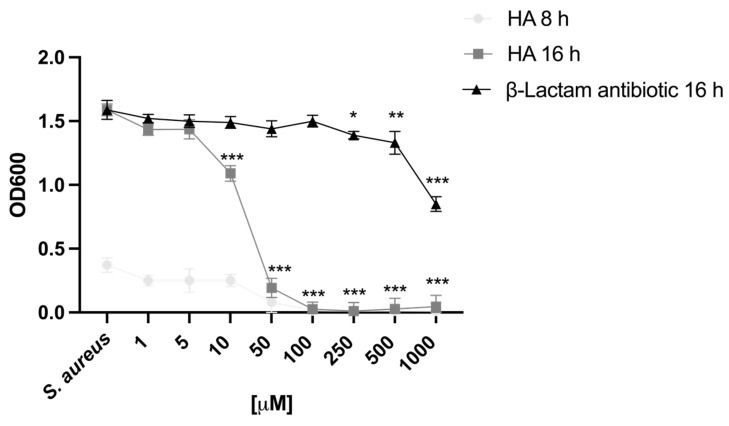
HA impacts *S. aureus* cell growth. *S. aureus* was incubated in the presence of LB medium as general control (*S. aureus*) or a range of HA (from 1000 μM to 1 μM). Growth curves were measured at an optic density of 600 nm (OD600) after 8 and 16 h of incubation. Β-lactam antibiotic was used as positive control. Statistical analysis was performed by two-way ANOVA, followed by Bonferroni correction test. Each condition was compared with the untreated control of the corresponding timepoint (*, *p <* 0.05; **, *p* < 0.01; ***, *p* < 0.001).

To determine whether HA affects *S. aureus* growth, the bacterial growth dynamic was evaluated for 16 h. HA was found to completely inhibit the growth of *S. aureus* at the highest tested concentrations (100 μM to 1000 μM) (Figure 1). Interestingly, bacterial growth suppression did not decrease over time (Figure 1). Of note, HA was also found to exert a modest antimicrobial activity against *S. aureus* at concentrations of 10 and 50 μM. In particular, a gradual and significant decrease in *S. aureus* growth was observed at 16 h of incubation with HA (10 and 50 μM), compared with that of untreated cells (*p* < 0.001) (Figure 1). Attractively, HA showed improved activity against *S. aureus* at 16 h compared with ampicillin, one of the most commonly used broad-spectrum antibiotics. Although the mechanisms underlying the suppression of bacterial growth following HA treatment remain to be determined, as a tetramic acid, HA might exert its antimicrobial activity by impairing the barrier function of the bacterial membrane. High concentrations of HA have been reported to produce pores in the cell membrane of Gram-positive bacteria [32]. However, the mechanism of pore formation is still unclear, but not depending on a direct targeting of the cell membrane. In fact, lacking a highly lipophilic N–substituent, HA seems not to be as effective in penetrating bacterial cell membranes. Generally, tetramic acid molecules possess lipophilic functional groups, exhibiting effective antibacterial activity. This property reflects the capability of these functional groups to (i) dissipate the transmembrane pH of bacteria, (ii) allow proton translocation across the membrane, and (iii) destroy the bacterial cell membrane [33]. In the absence of cell selectivity (prokaryotic cells over eukaryotic cells), it seems evident that tetramic acids also exhibit cytotoxic effects on eukaryotic cells [33]. Many studies, in fact, have focused on synthesizing novel molecules with reduced lipophilicity in order to improve their safety and preserve their antimicrobial effects. Yet, contrary to other tetramic acid derivatives, HA was found to be highly tolerated by eukaryotic cells, specifically HaCaT cells (See Section 2.2). This could find an explanation in its peculiar chemical structure. In such conditions, the capability of HA to chelate Fe^3+^ may represent an effective mechanism hampering bacterial iron availability, thus altering bacterial growth [29].

### 2.2. Harzianic Acid Does Not Alter HaCaT Cell Viability

**Figure 2:**
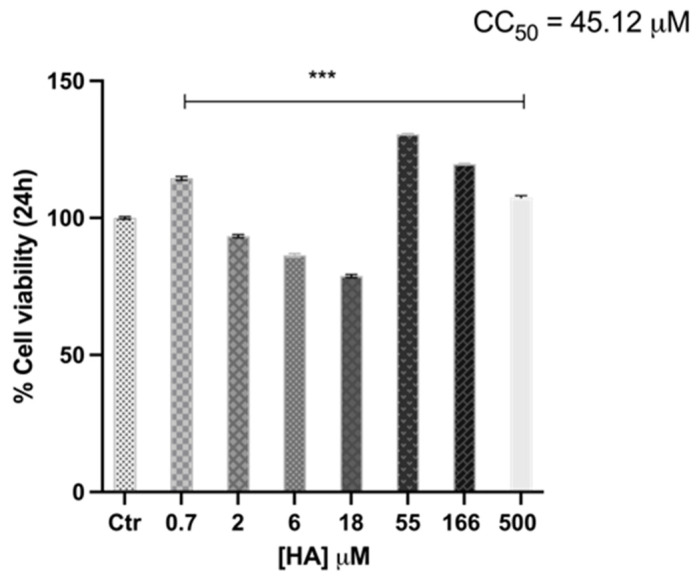
Effect of HA on HaCaT cell viability. HaCaT cells were cultured with different concentrations of HA for 24 h. The bar graph represents means ± SD of three independent experiments, each performed in triplicate. Results are expressed as percentage of untreated control. Statistical significance was determined by one-way ANOVA (***, *p* < 0.001). Statistical analysis was performed by comparing each condition with the untreated control. CC50, indicating cytotoxic concentration 50, was calculated by Graphad software.

To investigate whether HA affects the metabolic activity of human keratinocytes, the cytotoxicity of HA on HaCaT cells was explored. Despite different concentrations of HA were examined (spanning from 0.7 to 500 μM), no cytotoxic effects were detected (Figure 2). However, an unusual bimodal distribution was observed.

Of note, HaCaT cells treated with high concentrations of HA (55 to 500 μM) displayed an increased survival, compared with untreated cells (control; cell without HA treatment) (Figure 2). Yet, this phenomenon was not dependent on the HA dose.

Conversely, concentrations of HA lower than 55 μM decreased cell viability in a dose-dependent manner. However, cell viability was constantly more than 80% compared with the untreated control (Figure 2).

### 2.3. Harzianic Acid Controls the Cell Host Ca^2+^ Movements

Calcium signalling regulates diverse biological processes. Impairment of cellular calcium levels may alter cell metabolism and cause various diseases. The reasons of such alteration are multiple. It is generally reported that bacterial infections affect Ca^2+^ fluxes in host cells [34].

*Staphylococcus aureus* is recognized as the primary cause of skin infections [35]. It is described to invade and survive within the host cells for a long period of time, establishing a persistent infection [36]. Virulence factors, as well as its capability to escape the host immune defence, contribute to the pathogenicity and occurrence of *S. aureus* infection [37].

*Staphylococcus aureus* produces a variety of virulence factors, which may interfere with the host calcium signalling [13]. To invade the eukaryotic host cells, *S. aureus* produces pore-forming toxins, such as α–hemolysin, which induce Ca^2+^ oscillations in host cells, thus ensuring bacterial virulence and facilitating bacterial adaptation to the host environment [13,38,39].

**Figure 3:**
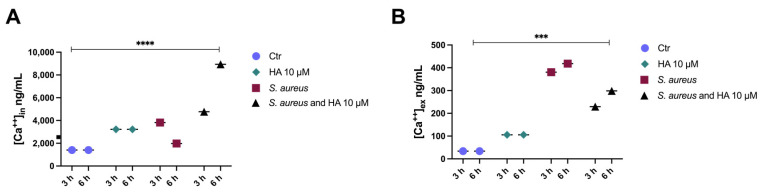
HA affects *S. aureus*-induced Ca^2+^ mobilization. HaCaT cells were infected with *S. aureus* or treated with HA 10 μM (after 90 min of bacterial exposure; *S. aureus* and HA), and cytosolic (**A**) as well as extracellular Ca^2+^ (**B**) were determined by Atomic Adsorption Spectroscopy after 3 or 6 h. Results are represented as means ± SD of three independent experiments, each performed in triplicate. Statistical significance was determined by one-way ANOVA followed by Bonferroni correction test (***, *p*< 0.001; ****, *p* < 0.0001). Statistical analysis was performed by comparing the above-reported experimental conditions (see legend) for each time of treatment.

Consistent with these findings, our results showed increased Ca^2+^ levels in HaCaT cells ([Ca^2+^]_in_) cultured with *S. aureus*. Interestingly, cells infected with *S. aureus* for 3 h responded to bacterial invasion with a higher rise of cytosolic calcium than those infected for 6 h (Figure 3A). The long-term exposure to *S. aureus* could account for this dissimilarity. To establish bacterial infection, *S. aureus* must colonize the host cells and replicate [36]. Nevertheless, 6 h post-infection, in vitro bacterial replication could cease, and the stationary growth phase could be reached, thus attenuating bacterial virulence and calcium increase.

Intracellular calcium elevation ([Ca^2+^]_in_) has also been reported to exert antimicrobial activity. In particular, Ca^2+^ ions have been demonstrated to kill stationary-phase *S. aureus* cells [40]. [Ca^2+^]_in_ in cells cultured with *S. aureus* for 6 h and treated with HA (10 µM) was increased when compared with both control and *S. aureus* cultured cells (Figure 3A). Such a result supports what is reported above and suggests that HA may mediate antimicrobial effects by targeting bacterial pathogens (at a high concentration; see Figure 1) and/or mammalian host cells, influencing the host immune responses [34].

Cells infected with *S. aureus* for a shorter time (3 h) and then treated with HA also showed an increased [Ca^2+^]_in_ (Figure 3A), likely due to the ability of HA to regulate the host’s innate immune response [41]. However, calcium accumulation was lower than in cells infected for 6 h (Figure 3A).

Bacterial infections also impact the extracellular calcium concentration ([Ca^2+^]_ex_) [42]. Furthermore, intracellular calcium accumulation often results in the extracellular calcium increase via transmembrane calcium fluxes. Nevertheless, extracellular calcium elevation may compromise the surrounding cell functions, triggering an exacerbated immune response through the NLRP3 inflammasome activation [42]. Intriguingly, HA was found to mitigate [Ca^2+^]_ex_ following *S. aureus* infection, compared with untreated cells (Figure 3B).

Taken together, these results indicate the valuable ability of HA to control *S. aureus* infections, by exerting a microbicidal action and/or supporting the host immune function against the pathogen, as well as to prevent a harmful inflammatory response.

**Figure 4:**
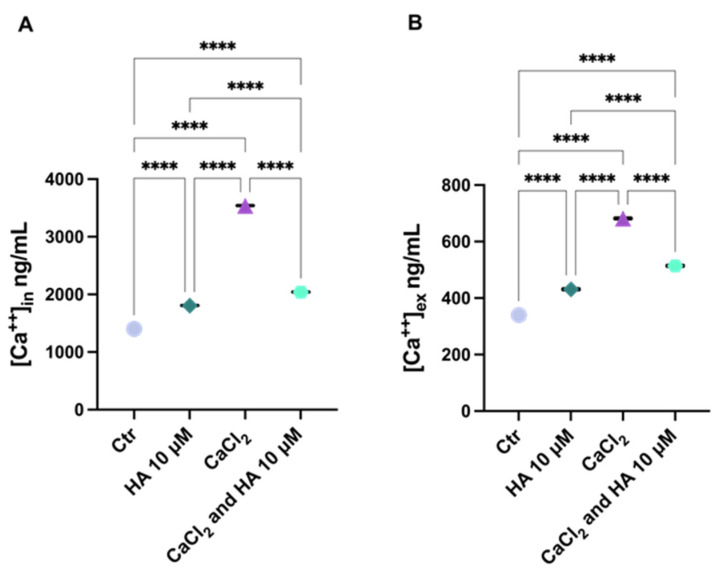
HA reduces CaCl_2_-induced Ca^2+^ oscillation. HaCaT cells were cultured with CaCl_2_ 1.8 mM or treated with HA 10 μM following 60 min of CaCl_2_ exposure. (**A**) Cytosolic Ca^2+^ and (**B**) extracellular Ca^2+^ levels were determined by colorimetric assay. Results are represented as means ± SD of three independent experiments, each performed in triplicate. Statistical significance was determined by one-way ANOVA followed by Bonferroni correction test (****, *p* < 0.0001).

In an attempt to investigate whether HA could hamper the host cell calcium oscillations, the culture medium of HaCaT cells was enriched with CaCl_2_ and the extracellular Ca^2+^ levels were measured following HA treatment, using a colorimetric method. Compared with control cells, calcium levels were increased in the culture medium of cells supplied with CaCl_2_ and, as expected, decreased in both supernatant and cytosol of cells treated with HA (Figure 4), indicating the capacity of HA to remove calcium ions—likely due to its chelating properties—and control cell calcium fluxes.

Therefore, the results revealed that HA may interfere with Ca(II) mobilization, not only in response to bacterial infections.

### 2.4. Coordination Properties of Harzianic Acid toward Ca^2+^

Harzianic acid has been widely recognized as an efficient ligand of a variety of metal cations [30,31,43,44]. Since harzianic acid is a diprotic acid, the symbol H_2_L is used in this section to indicate the fully protonated species, while HL^−^ and L^2−^ indicate deprotonated species whose dissociation constants were previously determined at 25 °C in 0.1 M NaClO_4_/(CH_3_OH + H_2_O 50/50 *w*/*w*) mixed solvent, the same solvent employed in this study [30].

Complex formation equilibria between harzianic acid toward the dipositive cation Ca^2+^ were studied using mass spectrometric, potentiometric, spectrophotometric, and nuclear magnetic resonance (NMR) techniques. A high-resolution mass spectrum (HRMS) of a solution consisting of CaCl_2_ and HA was acquired (Section 4.11). The most abundant ions in the collected HRMS are reported in Table 1. Peaks corresponding to adducts of harzianic acid with hydrogen, sodium, and potassium were detected. Moreover, peaks related to ions containing both Ca^2+^ cation and harzianic acid in a 1:2 and 1:3 metal-to-ligand ratio were observed.

**Figure 5:**
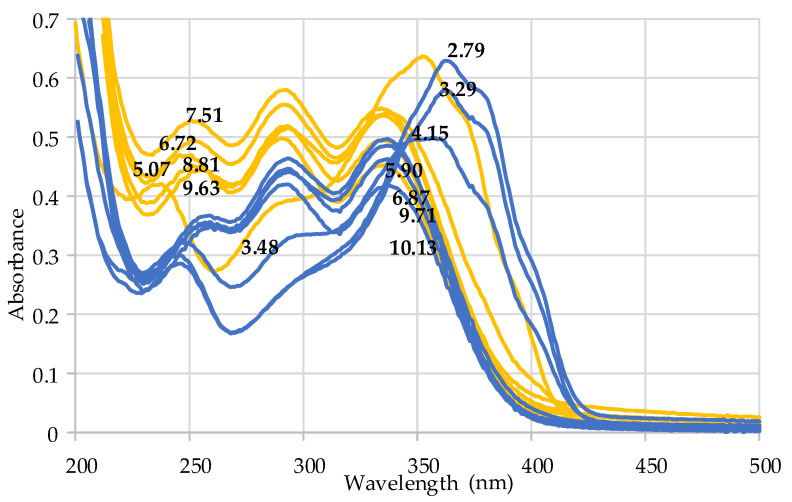
Raw UV–Vis spectra of solutions of Ca^2+^ and harzianic acid of accurately known analytical composition and pH in a 0.1 M NaClO_4_/(CH_3_OH + H_2_O 50/50 *w*/*w*) mixed solvent. Absorption spectra define two groups differentiated by color; orange-colored spectra have been acquired on solutions with a concentration of the ligand nearly twice that of the calcium dipositive cation; blue-colored spectra have been acquired on solutions with concentration of ligand nearly twice that of the metal cation. Numerical labels on curves indicate the corresponding pH.

The chelating properties of HA towards the dipositive cation Ca^2+^ were also studied by collecting potentiometric and spectrophotometric data. In particular, the interaction between harzianic acid and calcium cations was monitored by acquiring UV–Vis spectra in a wide wavelength range (200–500 nm) at 25 °C of solutions of accurately known analytical concentrations of the metal cation ($C_{\mathrm{Ca}}$, M) and of the ligand ($C_{H_{2}L}\;M$) in a 0.1 M NaClO_4_/(CH_3_OH + H_2_O 50/50 *w*/*w*) mixed solvent. The free proton concentration, [H^+^], was measured with a pH indicator glass electrode that was properly calibrated as is described in Materials and Methods, Section 4.12. By recording UV–Vis spectra as a function of pH at two molar metal/ligand ratios (Figure 5), it is evident that the spectral variations depend on the pH rather than on the molar metal/ligand ratio.

In order to evaluate the stoichiometry and formation constants between calcium metal ion and harzianic acid, the spectrophotometric data in Figure 5 were processed numerically by a Hyperquad program and the results are summarized in Table 2 [45].

**Figure 6:**
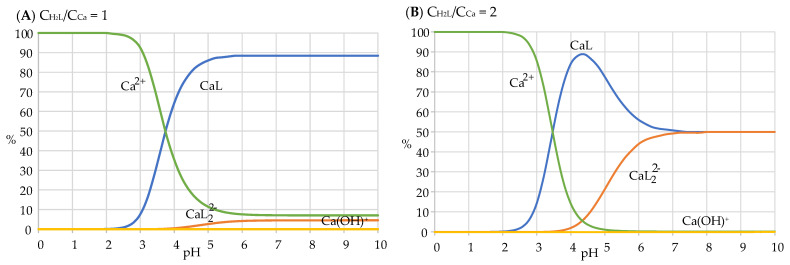
Distribution diagrams for Ca^2+^–harzianic acid systems in the 0.1 M NaClO_4_/(CH_3_OH + H_2_O 50/50 *w*/*w*) mixed solvent: (**A**) equal total concentrations (2.5 × 10^−4^ M) of harzianic acid and metal cations; (**B**) harzianic acid total concentration (5 × 10^−4^ M) twice the total concentration of metal cations.

From the data in Table 2, distribution diagrams can be drawn (Figure 6A,B) showing the fraction of the total calcium concentration present in the form of each species (free Ca^2+^ or complexed CaL/CaL_2_^2−^) as a function of pH. Either in solutions containing equal concentrations of the cation and of the ligand (i.e., $C_{H_{2}L}/C_{\mathrm{Ca}}=1$, Figure 6A) or in solutions in which the ligand concentration is twice the concentration of the cation (i.e., $C_{H_{2}L}/C_{\mathrm{Ca}}=2$, Figure 6B), it can be seen that, at low pH, the prevailing species in the solution is the free Ca^2+^ due to the protonation of the bonding sites of harzianic acid [40]. At higher pH values, the mono complex CaL rises to about 90% when the $C_{H_{2}L}/C_{\mathrm{Ca}}=1$, as in Figure 6A. Moreover, the bis complex CaL_2_^2−^ is present in negligible concentration (about 5%).

In solutions in which the ligand concentration is twice the concentration of the cation (i.e., $C_{H_{2}L}/C_{\mathrm{Ca}}=2$, Figure 6B), the mono complex CaL is the prevailing species at pH ≈ 4, while at the highest pH investigated (pH > 7), the bis complex and mono complex species are equally present in solution.

**Figure 7:**
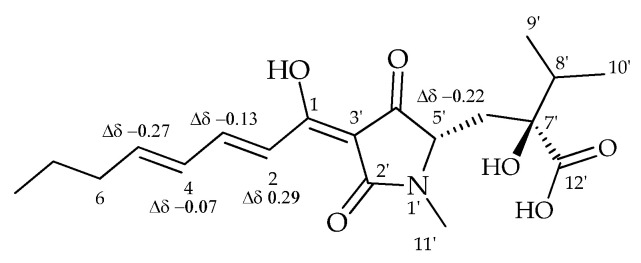
Main variations in chemical shifts observed by comparison of the ^1^H NMR of harzianic acid and Ca^2+^-harzianic acid complex recorded in CD_3_OD at 400 MHz.

NMR analysis of Ca^2+^–HA solutions confirmed the chelating ability of the fungal metabolite. In fact, the comparison of the proton spectra of harzianic acid and the solution of Ca^2+^ and harzianic acids recorded in CD_3_OD showed some significant shifts for the protons of the octadienoyl chain. In particular, the proton H–2, overlapped with H–3, resonated as a multiplet at δ 7.48–7.34, showing an upfield shift of Δδ 0.29. Moreover, protons H–3, H–4, and H–5 showed a downfield shift of Δδ 0.13, 0.07, and 0.27, respectively. The H–5’ proton of the pyrrolidine–2,4–dione ring resonated as a multiplet at δ 3.64–3.59, showing a downfield shift of Δδ 0.22. These data agree with those previously reported (Figure 7) [30,31].

## 3. Conclusions

In this investigation, the effects of the fungal metabolite harzianic acid were evaluated against *S. aureus,* a causative agent of severe infections, especially in hospitalized patients. Harzianic acid activity was tested towards human keratinocytes infected with *S. aureus*. The results provide evidence about the capability of HA in controlling *S. aureus*, reducing the pathogen growth rate, and modulating the eukaryotic cell Ca^2+^ mobilization. Furthermore, the capability of HA to complex calcium divalent cations was investigated using mass spectrometric, potentiometric, spectrophotometric, and NMR techniques. The results demonstrate the ability of this natural compound to form stable neutral or negatively charged complexes in a calcium/harzianic acid ratio 1:1 or 1:2. The abundance of the species is dependent on the pH of the solution.

In conclusion, HA may modulate the host Ca^2+^ signaling pathway, thus showing beneficial effects, which could have implications in different diseases. However, further studies are needed to investigate the mechanisms underpinning the ability of HA to modulate the host immune response against *S. aureus* via Ca^2+^ mobilization interference. Moreover, the comprehensive understanding of HA characteristics as a chelator of multiple metal ions could provide new insight into the therapeutical role of HA in *S. aureus* infections, highlighting its clinical (beneficial and adverse) effects.

## 4. Materials and Methods

### 4.1. Harzianic Acid Production

Harzianic acid was obtained from *T. harzianum* M10 grown in liquid medium (PDB, HiMedia, Mumbai, India) for 21 days and purified following a previously described protocol with some modifications [25]. Briefly, culture filtrate was exhaustively extracted with ethyl acetate (EtOAc, Carlo Erba, Cornaredo, Milan, Italy), and the dry residue was resuspended in dichloromethane (DCM, Carlo Erba) and extracted with a 2M solution of sodium hydroxide (NaOH, Carlo Erba). The aqueous phase was acidified at pH = 2 with hydrochloric acid (HCl, Carlo Erba), and harzianic acid was obtained after vacuum filtration and precipitate wash with EtOAc. Harzianic acid identification was achieved by NMR and LC-MS analyses [30,31].

### 4.2. Cell Culture Conditions

Immortalized human keratinocytes (HaCaT cell line) were obtained from the Cell Lines Service (CLS, catalog number 300493). HaCaT cells were grown in Dulbecco’s Modification of Eagle’s Medium, high glucose (DMEM), supplemented with 10% fetal bovine serum (FBS), 1% penicillin/streptomycin, and 1% L–glutamine (all from Microtech, Naples, Italy) in a humidified atmosphere at 37 °C and 5% CO_2_.

### 4.3. Staphylococcus aureus Growth Conditions

Starting from a frozen stock (−80 °C), *S. aureus* (ATCC 25923) was grown on Luria–Bertani (LB, Scharlab S.L., Barcelona, Spain) agar at 37 °C for 24 h. The next day, an isolated colony was transferred from the agar plate to the tube containing liquid LB medium. The tube was gently shaken to suspend bacterial cells and then incubated at 37 °C overnight on a shaker at 180 rpm.

### 4.4. Antibacterial Activity

Antimicrobial activity of HA against *S. aureus* was tested using the broth microdilution method [46]. HA was resuspended in LB medium containing 10% dimethyl sulfoxide (DMSO) and then diluted to obtain final concentrations used for the assay. In detail, four two-fold serially diluted concentrations of HA (1000 µM, 500 μM, 250 μM, 50 μM, and 5 μM) and three intermediate concentrations (100 μM, 10 μM, and 1 μM) were prepared in 15 mL sterile test tubes, using *S. aureus* growth medium (LB medium) as solvent. A final volume of 1 mL of each dilution (two times more concentrated than the above reported concentrations) was prepared using growth medium (LB) as diluent. Subsequently, the bacterial inoculum was prepared. In detail, the overnight culture was sub-cultured in fresh LB broth in order to reach the final concentration of 5 × 10^5^ CFU mL^–1^. An amount of 1 mL of the adjusted microbial suspension was added to each tube containing 1 mL of the sample and mixed. Finally, tubes were incubated at 37 °C on a shaker at 180 rpm for 16–18 h. The optical density at 600 nm (OD_600_) was measured using a spectrophotometer (Smartspec Plus, Bio-Rad, Hercules, CA, USA). Bacterial culture grown in LB medium containing 10% DMSO and in the absence of HA was used as control, while LB medium containing 10% DMSO was used as blank.

### 4.5. Cell Viability Assay

Colorimetric MTT assay was performed to examine the cytotoxic effect of HA on HaCaT cells [47]. Cells were seeded in a 96-well plate at a density of 2 × 10^5^ per well and incubated overnight at 37 °C and 5% CO_2_. HA was resuspended in a mixture of cell culture medium (DMEM) and 10% DMSO, generating the stock solution. The stock solution was successively diluted, using cell culture medium, in order to prepare HA solutions at various concentrations used in the test (0.7–500 μM). The next day, culture medium was replaced with fresh medium containing different concentrations of HA (0.7–500 µM) and cells were further incubated for 24 h. After the incubation time, the medium containing the treatment under investigation was removed by aspiration, cells were washed with PBS, and fresh culture medium diluted with 3–(4, 5–dimethylthiazolyl–2)–2, 5–diphenyltetrazolium bromide solution (MTT; 1:10) was added to each well. Cells were further incubated for 3 h at 37 °C and 5% CO_2_. The formed crystals were dissolved using DMSO and quantified using the Model 680 microplate reader (Bio-rad, Hercules, CA, USA) at 570 nm. The percentage of cell viability was calculated according to the following formula: (ABS_sample_ − ABS_blank_)/(ABS_control_ − ABS_blank_) × 100, where blank represents cells incubated with 10% DMSO in culture medium and control represents untreated cells. CC_50_ was calculated by GraphPad Prism software (version 9.1.1) using nonlinear regression analysis.

### 4.6. Infection by Staphylococcus aureus of Eukaryotic Cells

Infection of cells was performed following Stelzner et al. (2020) protocol with some modifications [36]. The day before the infection, 0.25 × 10^6^ cells were seeded in a 24-well plate and incubated overnight at 37 °C and 5% CO_2_. The next day an OD_600_ = 0.4 cell suspension was incubated at 37 °C for 1 h on a shaker at 180 rpm. When the exponential growth phase was reached, bacteria were centrifugated twice, at 6000× *g* for 10 min, and washed with Phosphate Buffer Solution (PBS; Microtech, Naples, Italy). Bacterial cells were then resuspended in DMEM with or without 10 μM HA and used to infect HaCaT cells (Multiplicity of Infection; MOI = 50). After 1 h of infection, extracellular bacteria were removed by lysostaphin treatment (20 μg mL^−1^ per well for 30 min). Finally, culture medium was removed, and cells were washed in PBS and restored with fresh medium containing 10 μM HA. After an additional 2 or 5 h of incubation, both culture medium and cells were collected and stored at –80 °C until calcium measurement.

### 4.7. Extracellular Ca^2+^ Supplementation

HaCaT cells were supplied with exogenous Ca^2+^, to better investigate the role of HA in controlling calcium oscillations. Cells were seeded in a 24-well plate at a density of 0.25 × 10^6^ per well and incubated at 37 °C and 5% CO_2_ overnight. After cell attachment, the culture medium was renewed with a fresh one containing 1.8 mM CaCl_2_ [36], with or without 10 μM HA, and incubated for 1 h. After the incubation time, cells were treated with 10 μM HA and further incubated. At 2 h after treatment, both culture medium and cells were collected and stored at –80 °C until calcium measurement.

### 4.8. Ca^2+^ Measurement by Atomic Adsorption Spectroscopy

Intracellular and extracellular calcium content was determined by Atomic Adsorption Spectroscopy, as reported by Fiorito et al. in 2021 [48]. For the analysis, an AA–6300 spectrophotometer (Shimadzu, Columbia, MD, USA) equipped with an ASC–6100 autosampler (Shimadzu, Columbia, MD, USA) and a GFA–EX7i graphite furnace atomizer (Shimadzu, Columbia, MD, USA) was used. Prior the analysis, a fine mist dispersion of cell culture medium or cell pellets was prepared, using a microwave digestion apparatus (MW–AD, Ethos EZ microwave digester, Mileston, Shelton, CT, USA). Samples were transferred into TFM^®^PTFE vessels, and 6 mL of ultra-pure concentrated HNO_3_ (14.33 mol L^−1^) and 1 mL of 30% H_2_O_2_ were added. The heating program for digestion was 160 °C for 5 min using 80% of microwave power, 190 °C for 10 min using 90% of microwave power, 50 °C for 11 min. Final solutions were diluted up to 25 mL with water and mineralized at 550 °C for 4 h. The analyte was detected according to the following working conditions: wavelength, 248.3 nm; slit width, 0.5 nm; lamp current, 5 mA; gas, Argon.

### 4.9. Ca^2+^ Measurement by Colorimetric Method

The concentration of free calcium ions was determined in order to assess whether HA can chelate cell Ca^2+^, by colorimetric assay, using a calcium assay kit (Abcam, Cambridge, UK, #ab102505). Following Ca^2+^ supplementation and HA treatment (as reported above, Section 4.7), calcium concentration in both cells and cell medium was measured according to the manufacturer’s instructions. Briefly, after sample preparation, the reaction mixture was added and incubated at room temperature for 5–10 min protected from light. The optical density was measured at 575 nm using a microplate reader (Model 680, Bio-rad, Hercules, CA, USA).

### 4.10. Reagents and Their Analysis

Stock solutions of calcium perchlorate for spectrophotometric and NMR measurements were prepared by dissolving its high-purity calcium carbonate anhydrous in concentrated perchloric acid (Merck, Darmstadt, Germany). The solution obtained was brought to a boil to remove the carbon dioxide produced, and then it was cooled. The exact concentration of metal solution was determined as described by Kolthoff et al. 1978 [49].

NMR spectra were recorded at 400 MHz in CD_3_OD on a Bruker spectrometer (Ascend^TM^400) (Bremen, Germany). The solvent was used as internal standard.

UV–Vis spectra were recorded by Cary model 5000 Spectrophotometer by Varian C. (Palo Alto, CA, USA), from 200 to 600 nm (optical path 0.2 cm) at 25.0 °C and under a constant flow of nitrogen.

### 4.11. HPLC-ESI-Q-TOF Analysis

HPLC-ESI-Q-TOF analysis was carried out on a quadrupole time-of-flight (Q–TOF) mass spectrometer (Agilent Technologies, Santa Clara, CA, USA), equipped with a Dual electrospray ionization source (Agilent Technologies) and coupled to a 1260 Infinity Series high-performance liquid chromatograph (Agilent Technologies). A solution consisting of CaCl_2_ (2 mM, aqueous solution) and harzianic acid (1 mg mL^−1^, methanolic solution) in a 1:1 (*v*/*v*) ratio was directly infused into the LC system. Elution, spectral, and all instrumental parameters were set following the method described by De Tommaso et al. [30]. Acquisition was achieved using Agilent MassHunter Data Acquisition Software, rev. B.05.01 (Agilent Technologies).

### 4.12. Preparation of Test Solutions for UV–Vis Spectrophotometric Measurements

The UV–Vis measurements of solutions of calcium cations and harzianic acid required the acquisition of spectra of solutions of accurately known pH (= −log[H^+^]); analytical compositions of metal ion $C_{\mathrm{Ca}}$ M, HA $C_{H_{2}L}$ M, $C_{H}$ M (analytical concentration of HClO_4_); and $C_{\mathrm{OH}}^{\;}$ M (analytical concentration of NaOH) in the 0.1 M NaClO_4_/(CH_3_OH + H_2_O 50/50 *w*/*w*) mixed solvent. In particular, the spectra were collected to have the same ligand-to-metal ratio (i.e., $C_{H_{2}L}/C_{\mathrm{Ca}}$= *constant*). The free hydrogen ionic concentration was measured with a potentiometric apparatus constituted by a multi-neck titration vessel equipped with a Metrohm AG (Herisau, Switzerland) 60102–100 pH sensitive glass electrode (GE) and an Ag/AgCl_(s)_/0.1 M NaCl/(0.1 M NaClO_4_/(CH_3_OH + H_2_O 50/50 *w*/*w*) double-junction reference electrode (RE).

The experiment started by introducing a fixed volume, $V_{H}$ mL, of the HClO_4_ stock solution in the titration vessel, which was kept in an air thermostat at 25 °C ± 0.1 °C. This realized a potentiometric cell, GE/Solution/RE, whose potential, $E_{G}\;\mathrm{Volt}$, under the present conditions can be expressed by the following relation (1):

(1) $$E_{G}\left(\mathrm{Volt}\right)=E_{G}^{0}\left(\mathrm{Volt}\right)+Slope\cdot \log\left[H^{+}\right]$$

The calibration constants, $E_{G}^{0}$, and *Slope* in Equation (1) were evaluated by preparing a solution in the potentiometric vessel, which was alkalimetrically titrated by stepwise addition of accurately measured volumes of the $C_{\mathrm{OH}}^{0}$ M stock solution of NaOH. The alkalimetric titration ended when the same total volume, $V_{\mathrm{OH}}$, of NaOH solution was added and the solution in the potentiometric vessel attained a fixed volume equal to $\left(V_{H}+V_{\mathrm{OH}}\right)$ mL. After this titration, accurately measured volumes of the $C_{\mathrm{Ca}}^{0}$ M solution of Ca^2+^ and of the $C_{H_{2}L}^{0}$ M solution of harzianic acid were added to the $\left(V_{H}+V_{\mathrm{OH}}\right)$ mL of solution in the titration vessel. The added volumes of harzianic acid and metal solutions determined the ligand-to-metal ratio in the resulting solution. Hence, the solution was brought to its final pH by adding a measured volume of the $C_{\mathrm{OH}}^{0}$ M solution of NaOH. The volume of the added NaOH solution determined the values of $C_{\mathrm{Ca}}$, $C_{H_{2}L},$ and pH in the final solution, which was used for UV–Vis analyses; it did not change the ligand-to-metal ratio.

Subsequently, sufficient time was allowed for chemical equilibrium to be established and for the glass electrode potential, $E_{G}$, to achieve a constant value, which persisted for at least 15 min within ± 0.1 mV. Thus, the free proton concentration of the solution in the titration vessel was readily calculated from Equation (2) and the measured $E_{G}$, as follows:

(2) $$E_{G}=E_{G}^{0}+Slope\cdot \log\left[H^{+}\right]\to \;\mathrm{pH}=-\log\left[H^{+}\right]=\frac{E_{G}^{0}-E_{G}}{Slope}\;$$

Finally, appropriate volumes of solution were withdrawn from the titration vessel and submitted UV–Vis spectrophotometer at 25.0 °C.

In this way, using fixed volumes of the $C_{\mathrm{Ca}}^{0}$ M stock solution of Ca^2+^ and the $C_{H_{2}L}^{0}$ M stock solution of HA for each group of UV–Vis measurements, the ratio $C_{H_{2}L}/C_{\mathrm{Ca}}$ was kept the same in each group.

Spectrophotometric data were processed numerically by the Hyperquad program [45] to evaluate the stoichiometry and formation constants between the calcium metal ion and HA. The program for equilibrium data interpretation fits the experimental data by systematically modifying the equilibrium constants of an assumed set of species to minimize the sum of squared weighted residuals (U). In Equation (3), A_iK_ represents the absorbance measured at the k*–th* wavelength for the i*–th* solution, (A_iK_)_c_ is the absorbance calculated for a fixed set of equilibrium constants, and the wk values are the weights assigned to each measurement. In the present work, we have assumed wk = 1.

(3) $$U=\sum_{i}\sum_{k}{\left(A_{ik}-A_{ik}^{c}\right)}^{2}$$

### 4.13. Statistical Analysis

Statistical analysis was performed using GraphPad Prism Software version 9.1.1 (San Diego, CA, USA). Multiple comparisons were carried out using one-way or two-way ANOVA, followed by Bonferroni correction test. One-way ANOVA was used to compare two or more experimental conditions, while two-way ANOVA was used to compare two or more experimental conditions and two variables (factors). Data are represented as means ± SD resulting from three biological replicates and are considered statistically significant when *p* value is < 0.05.

## Figures and Tables

**Table 1:** Most abundant ions in high-resolution spectrum (HRMS) acquired by HPLC-ESI-HRMS on solutions of harzianic acid and Ca^2+^.

Ion	Experimental Mass of Main Isotopic Peak (Da)	Formula	Exact Mass (Da)
Harzianic acid + CaCl_2_			
[H_2_L + H]^+^	366.1929	C_19_H_28_NO_6_	366.1917
[H_2_L + Na]^+^	388.1750	C_19_H_27_NO_6_Na	388.1736
[H_2_L + K]^+^	404.1404	C_19_H_27_NO_6_K	404.1475
[2H_2_L − H + Ca]^+^	769.3243	C_38_H_53_N_2_O_12_Ca	769.3224
[3H_2_L − H + Ca]^+^	1134.5079	C_57_H_80_N_3_O_18_Ca	1134.5063

**Table 2:** Summary of Ca^2+^/harzianic acid (H_2_L) formation constants. σ indicates the estimated standard deviation.

Equilibria	log (Formation Constant) ± 3σ
Ca^2+^ + L^2−^ = CaL Ca^2+^ + 2L^2−^ = CaL_2_^2−^	6.3 ± 0.1 10.2 ± 0.1

## Data Availability

The data that support the findings of this study are available from the corresponding author upon reasonable request.

## Sample Availability

Samples of harzianic acid are available from the authors.

## References

[1] Berridge M.J., Lipp P., Bootman M.D. (2000). The versatility and universality of calcium signaling. Nat. Rev. Mol. Cell.

[2] Edel K.H., Kudla J. (2015). Increasing complexity and versatility: How the calcium signaling toolkit was shaped during plant land colonization. Cell Calcium.

[3] Permyakov E.A., Kretsinger R.H. (2009). Cell signaling, beyond cytosolic calcium in eukaryotes. J. Inorg. Biochem..

[4] Clapham D.E. (1995). Calcium signaling. Cell.

[5] Bose J., Pottosin I.I., Shabala S.S., Palmgren M.G., Shabala S. (2011). Calcium efflux systems in stress signaling and adaptation in plants. Front. Plant Sci..

[6] Weiss N., Koschak A. (2014). Pathologies of Calcium Channels.

[7] Tran Van Nhieu G., Dupont G., Combettes L. (2018). Ca^2+^ signals triggered by bacterial pathogens and microdomains. Biochim. Biophys. Acta Mol. Cell Res..

[8] David M.Z., Daum R.S., Bagnoli F., Rappuoli R., Grandi G. (2017). Treatment of *Staphylococcus aureus* Infections. Staphylococcus aureus. Current Topics in Microbiology and Immunology.

[9] Tong S.Y., Davis J.S., Eichenberger E., Holland T.L., Fowler V.G. (2015). *Staphylococcus aureus* infections: Epidemiology, pathophysiology, clinical manifestations, and management. Clin. Microbiol. Rev..

[10] Olaniyi R., Pozzi C., Grimaldi L., Bagnoli F., Bagnoli F., Rappuoli R., Grandi G. (2016). Staphylococcus aureus-Associated Skin and Soft Tissue Infections: Anatomical Localization, Epidemiology, Therapy and Potential Prophylaxis. Staphylococcus aureus. Current Topics in Microbiology and Immunology.

[11] Peraro M., van der Goot F. (2016). Pore-forming toxins: Ancient, but never really out of fashion. Nat. Rev. Microbiol..

[12] Marchi S., Morroni G., Pinton P., Galluzzi L. (2022). Control of host mitochondria by bacterial pathogens. Trends Microbiol..

[13] Eichstaedt S., Gäbler K., Below S., Müller C., Kohler C., Engelmann S., Hildebrandt P., Völker U., Hecker M., Hildebrandt J.P. (2009). Effects of *Staphylococcus aureus*-hemolysin A on calcium signaling in immortalized human airway epithelial cells. Cell Calcium.

[14] Singh R., Kumar M., Mittal A., Mehta P.K. (2017). Microbial metabolites in nutrition, healthcare and agriculture. 3 Biotech.

[15] Ramírez-Rendon D., Passari A.K., Ruiz-Villafán B., Rodríguez-Sanoja R., Sánchez S., Demain A.L. (2022). Impact of novel microbial secondary metabolites on the pharma industry. Appl. Microbiol. Biotechnol..

[16] Atanasov A.G., Zotchev S.B., Dirsch V.M., Supuran C.T., International Natural Product Sciences Taskforce (2021). Natural products in drug discovery: Advances and opportunities. Nat. Rev. Drug Discov..

[17] Lucke M., Correa M.G., Levy A. (2020). The Role of Secretion Systems, Effectors, and Secondary Metabolites of Beneficial Rhizobacteria in Interactions with Plants and Microbes. Front. Plant Sci..

[18] Sinno M., Ranesi M., Di Lelio I., Iacomino G., Becchimanzi A., Barra E., Molisso D., Pennacchio F., Digilio M.C., Vitale S. (2021). Selection of Endophytic *Beauveria bassiana* as a Dual Biocontrol Agent of Tomato Pathogens and Pests. Pathogens.

[19] Harman G.E., Howell C.R., Viterbo A., Chet I., Lorito M. (2004). *Trichoderma* species—Opportunistic, avirulent plant symbionts. Nat. Rev. Microbiol..

[20] Vinale F., Sivasithamparam K., Ghisalberti E.L., Marra R., Woo S.L., Lorito M. (2008). Trichoderma–plant–pathogen interactions. Soil Biol. Biochem..

[21] Keswani C., Mishra S., Sarma B.K., Singh S.P., Singh H.B. (2014). Unraveling the efficient applications of secondary metabolites of various *Trichoderma* spp.. Appl. Microbiol. Biotechnol..

[22] Vinale F., Sivasithamparam K. (2020). Beneficial effects of *Trichoderma* secondary metabolites on crops. Phytother. Res..

[23] Ramírez-Valdespino C.A., Casas-Flores S., Olmedo-Monfil V. (2019). *Trichoderma* as a Model to Study Effector-Like Molecules. Front. Microbiol..

[24] Vinale F., Nigro M., Sivasithamparam K., Flematti G., Ghisalberti E.L., Ruocco M., Varlese R., Marra R., Lanzuise S., Eid A. (2013). Harzianic acid: A novel siderophore from *Trichoderma harzianum*. FEMS Microbiol. Lett..

[25] Vinale F., Flematti G., Sivasithamparam K., Lorito M., Marra R., Skelton B.W., Ghisalberti E.L. (2009). Harzianic acid, an antifungal and plant growth promoting metabolite from *Trichoderma harzianum*. J. Nat. Prod..

[26] Dini I., Pascale M., Staropoli A., Marra R., Vinale F. (2021). Effect of Selected *Trichoderma* Strains and Metabolites on Olive Drupes. Appl. Sci..

[27] Manganiello G., Sacco A., Ercolano M.R., Vinale F., Lanzuise S., Pascale A., Napolitano M., Lombardi N., Lorito M., Woo S.L. (2018). Modulation of tomato response to *Rhizoctonia solani* by *Trichoderma harzianum* and its secondary metabolite harzianic acid. Front. Microbiol..

[28] Dini I., Graziani G., Fedele F.L., Sicari A., Vinale F., Castaldo L., Ritieni A. (2020). Effects of *Trichoderma* Biostimulation on the Phenolic Profile of Extra-Virgin Olive Oil and Olive Oil By-Products. Antioxidants.

[29] De Filippis A., Nocera F.P., Tafuri S., Ciani F., Staropoli A., Comite E., Bottiglieri A., Gioia L., Lorito M., Woo S.L. (2021). Antimicrobial activity of harzianic acid against *Staphylococcus pseudintermedius*. Nat. Prod. Res..

[30] De Tommaso G., Salvatore M.M., Nicoletti R., DellaGreca M., Vinale F., Bottiglieri A., Staropoli A., Salvatore F., Lorito M., Iuliano M. (2020). Bivalent Metal-Chelating Properties of Harzianic Acid Produced by *Trichoderma pleuroticola* Associated to the Gastropod *Melarhaphe neritoides*. Molecules.

[31] De Tommaso G., Salvatore M.M., Nicoletti R., DellaGreca M., Vinale F., Staropoli A., Salvatore F., Lorito M., Iuliano M., Andolfi A. (2021). Coordination Properties of the Fungal Metabolite Harzianic Acid Toward Toxic Heavy Metals. Toxics.

[32] Ouyang X., Hoeksma J., Beenker W.A.G., van der Beek S., den Hertog J. (2021). Harzianic Acid Has Multi-Target Antimicrobial Activity against Gram-Positive Bacteria.

[33] Yendapally R., Hurdle J.G., Carson E.I., Lee R.B., Lee R.E. (2008). N-substituted 3-acetyltetramic acid derivatives as antibacterial agents. J. Med. Chem..

[34] King M.M., Kayastha B.B., Franklin M.J., Patrauchan M.A., Islam M. (2020). Calcium Regulation of Bacterial Virulence. Calcium Signaling. Advances in Experimental Medicine and Biology.

[35] McCaig L.F., McDonald L.C., Mandal S., Jernigan D.B. (2006). *Staphylococcus aureus*-associated skin and soft tissue infections in ambulatory care. Emerg. Infect. Dis..

[36] Stelzner K., Winkler A.C., Liang C., Boyny A., Ade C.P., Dandekar T., Fraunholz M.J., Rudel T. (2020). Intracellular *Staphylococcus aureus* perturbs the host cell Ca^2+^ homeostasis to promote cell death. mBio.

[37] Cheung G.Y.C., Bae J.S., Otto M. (2021). Pathogenicity and virulence of *Staphylococcus aureus*. Virulence.

[38] Menestrina G., Dalla Serra M., Comai M., Coraiola M., Viero G., Werner S., Colin D.A., Monteil H., Prévost G. (2003). Ion channels and bacterial infection: The case of beta-barrel pore-forming protein toxins of *Staphylococcus aureus*. FEBS Lett..

[39] Tengholm A., Hellman B., Gylfe E. (2000). Mobilization of Ca^2+^ stores in individual pancreatic β-cells permeabilized or not with digitonin or α-toxin. Cell Calcium.

[40] Xie Y., Yang L. (2016). Calcium and magnesium ions are membrane-active against stationary-phase *Staphylococcus aureus* with high specificity. Sci. Rep..

[41] Büchau A.S., Gallo R.L. (2007). Innate immunity and antimicrobial defense systems in psoriasis. Clin. Dermatol..

[42] Rossol M., Pierer M., Raulien N., Quandt D., Meusch U., Rothe K., Schubert K., Schöneberg T., Schaefer M., Krügel U. (2012). Extracellular Ca^2+^ is a danger signal activating the NLRP3 inflammasome through G protein-coupled calcium sensing receptors. Nat. Commun..

[43] De Tommaso G., Salvatore M.M., Siciliano A., Staropoli A., Vinale F., Nicoletti R., DellaGreca M., Guida M., Salvatore F., Iuliano M. (2022). Interaction of the Fungal Metabolite Harzianic Acid with Rare-Earth Cations (La^3+^, Nd^3+^, Sm^3+^, Gd^3+^). Molecules.

[44] Salvatore M.M., Siciliano A., Staropoli A., Vinale F., Nicoletti R., DellaGreca M., Guida M., Salvatore F., Iuliano M., Andolfi A. (2022). Interaction of the Fungal Metabolite Harzianic Acid with Rare-Earth Cations (Pr^3+^, Eu^3+^, Ho^3+^, Tm^3+^). Molecules.

[45] Gans P., Sabatini A., Vacca A. (1996). Investigation of equilibria in solution. Determination of equilibrium constants with the HYPERQUAD suite of programs. Talanta.

[46] Balouiri M., Sadiki M., Ibnsouda S.K. (2016). Methods for in vitro evaluating antimicrobial activity: A review. J. Pharm. Anal..

[47] Cuomo P., Medaglia C., Allocca I., Montone A.M.I., Guerra F., Cabaro S., Mollo E., Eletto D., Papaianni M., Capparelli R. (2021). Caulerpin Mitigates *Helicobacter pylori*-Induced Inflammation via Formyl Peptide Receptors. Int. J. Mol. Sci..

[48] Fiorito F., Irace C., Nocera F.P., Piccolo M., Ferraro M.G., Ciampaglia R., Tenore G.C., Santamaria R., De Martino L. (2021). MG-132 interferes with iron cellular homeostasis and alters virulence of bovine herpesvirus 1. Res. Vet. Sci..

[49] Kolthoff I.M., Elving P.J., Meehan E.J. (1978). Treatise on Analytical Chemistry.

